# Molecular Characterization and Pathogenicity Analysis of Porcine Rotavirus A

**DOI:** 10.3390/v16121842

**Published:** 2024-11-27

**Authors:** Yaning Lv, Ze Tong, Jiaqi Liu, Zhaoran Zhang, Chenchen Wang, Yan Zeng, Pingxuan Liu, Xin Zong, Guosheng Chen, Huanchun Chen, Chen Tan

**Affiliations:** 1 National Key Laboratory of Agricultural Microbiology, College of Veterinary Medicine, Huazhong Agricultural University, Wuhan 430070, China; 2 Key Laboratory of Preventive Veterinary Medicine in Hubei Province, The Cooperative Innovation Center for Sustainable Pig Production, Wuhan 430070, China

**Keywords:** porcine rotavirus A, genotype, intestines, phylogenetic analyses

## Abstract

Porcine rotavirus A (RVA) is one of the major etiological agents of diarrhea in piglets and constitutes a significant threat to the swine industry. A molecular epidemiological investigation was conducted on 2422 diarrhea samples from Chinese pig farms to enhance our understanding of the molecular epidemiology and evolutionary diversity of RVA. The findings revealed an average RVA positivity rate of 42% (943/2422), and the study included data from 26 provinces, primarily in the eastern, southern and southwestern regions. Genetic evolutionary analysis revealed that G9 was the predominant genotype among the G-type genotypes, accounting for 25.32% of the total. The VP4 genotypes were P[7] (36.49%) and P[23] (36.49%). The predominant genotypic combinations of RVA were G9P[23] and G9P[7]. Eleven RVA strains were obtained via MA104 cell isolation. A rat model was established to assess the pathogenicity of these strains, with three strains exhibiting high pathogenicity in the model. Specifically, the RVA Porcine CHN HUBEI 2022 (Q-1), RVA Porcine CHN SHANXI 2022 (3.14-E), and RVA Porcine CHN HUBEI 2022 (5.11-U) strains were shown to cause diarrhea in the rats and damage the intestinal villi during the proliferation phase of the infection, leading to characteristic lesions in the small intestine. These data indicate that continuous monitoring of RVA can provide essential data for the prevention and control of this virus.

## 1. Introduction

From 2018 to 2022, the prevalence of porcine rotavirus increased from 22.7% to 51.7%, with clinical manifestations of anorexia, vomiting, and watery diarrhea [1,2]. The prevalence of porcine rotavirus has been increasing and is a growing concern.

Porcine rotaviruses belong to the Reoviridae family, are nonenveloped, double-stranded RNA (dsRNA) viruses with a genome size of approximately 18.5 kb, consisting of 11 discrete linear segments [3]. The genome contains at least eleven major open reading frames (ORFs), encoding six structural proteins (VP1–VP4, VP6, and VP7) and five nonstructural proteins (NSP1–NSP6) [4]. The genome is encased in a three-layered icosahedral protein shell, and the virions are approximately 70 nm in diameter. The innermost capsid is composed of VP2, VP1, and VP3, which encapsulate the genome. The genomic nucleic acids are closely linked to the VP1, VP3, and VP2 proteins. VP1 and VP3 exhibit RNA transcriptase and modifier enzyme activities. The intermediate layer is composed of 260 conserved VP6 trimers. The outermost layer is composed of the VP7 and VP4 proteins, which are essential for viral invasion and are antigenically active to stimulate the secretion of neutralizing antibodies by the host [4]. Additionally, the nonstructural proteins are involved in viral genome replication, translation, and virion assembly [5]. Rotaviruses are classified into groups A through J. Rotavirus A, B, and C are capable of infecting both humans and animals; among these, Rotavirus A (RVA) is the predominant strain responsible for causing diarrhea in nursing piglets. According to genetic classification, the genome of Rotavirus A is further subdivided into distinct genotypes [6,7]. The well-known dual classification system classifies rotavirus genotypes mainly on the basis of the VP4 and VP7 proteins. In this system, the P-genotype of the virus is determined by the VP4 protein, and the G-genotype is determined by VP7 [8]. To date, rotaviruses have been classified into at least 27 G-genotypes and 37 P-genotypes on the basis of nucleotide sequence differences in the VP7 and VP4 capsid proteins [9]. However, this binary classification system tends to emphasize only the importance of VP4 and VP7 [10]. The Working Group on Rotavirus Classification proposed a new classification system for group A rotaviruses. This system is based on the homology of the entire genome (11 gene segments) and the homology thresholds of the different segments, denoted as Gx-Px-Ix-Rx-Cx-Mx-Ax-Nx-Tx-Ex-Hx. These represent VP7-VP4-VP6-VP1-VP2-VP3-NSP1-NSP2-NSP3-NSP4-NSP5/6, classified on the basis of nucleotide percent identity cutoff values of 80%, 80%, 85%, 83%, 84%, 81%, 79%, 85%, 85%, 85%, and 91% for each segment [11]. Since this method is based on all of the gene segments of rotaviruses, it enables further analysis of the host–range restriction, replication, and virulence genes, as well as the epidemiology and evolution of rotaviruses [12].

Porcine rotavirus A (RVA) has not received much attention because of its low mortality rate and tendency to cause only small-scale epidemics [13,14]. However, the re-emergence and rapid spread of RVA have been reported in recent years [15]. Notably, a serious outbreak of piglet diarrhea occurred in China’s pig-producing provinces, causing millions of neonatal piglet deaths due to diarrhea-related dehydration and weight loss [16]. The spread of RVA has caused significant economic losses in many pig-producing countries globally [17,18]. Additionally, the prevalence of RVA has accelerated the reorganization and mutation of its genome. RVA has several genotypes that are dominated by the G and P genotypes. The G genotypes range from G1 to G6, G8, G9, G11, G12, and G26, whereas the P genotypes include P[1] to P[8], P[11], P[13], P[19], P[23], and P[26] to P[28] [19]. Several studies have indicated that the predominant G genotypes currently prevalent in pigs are G9, G5, and G4, with fewer occurrences of G3, G11, and G26; similarly, the predominant P genotypes are P[23], P[7], and P[13], with fewer occurrences of P[5] and P[28] [20]. Rotavirus strains of the same genogroup of RVA can undergo genetic rearrangements, point mutations, reassortments, and recombination events in all 11 gene segments of RVA, leading to a large number of genotypes [21]. The diversity of RVA serotypes has led to more frequent occurrences of multisystem diseases, posing significant challenges to disease prevention and vaccine selection. This study investigated the positivity rate, genetic diversity, evolutionary relationships, and pathogenicity of porcine rotavirus A, aiming to provide valuable insights for enhancing its prevention and control strategies.

## 2. Materials and Methods

### 2.1. Sample Preparation and Detection

A total of 2242 diarrhea samples (taken directly from the small intestine) were collected from 150 different industrial pig farms across 26 provinces and cities in China between 2021 and 2024. The tissue shears used for each clipped sample were cleaned, disinfected with an alcohol cotton ball, and cauterized with the external flame of an alcohol lamp to prevent cross-contamination. Subsequently, 800 μL of PBS was added to each cryopreservation tube containing the small segmented intestinal tissue, followed by crushing the tissues with a tissue grinder. Total RNA was extracted from the collected samples after thawing and centrifugation using the TransZol Up Plus RNA extraction kit (TransGen, Beijing, China). A series of primers were designed to detect pathogens, including porcine epidemic diarrhea virus (PEDV), Transmissible gastroenteritis virus (TGEV), Porcine rotavirus A (RVA), and porcine deltacorona virus (PDCoV) using methods as previously described [22]. RT-qPCR was performed by using the TransScript Probe One-Step qRT-PCR Kit (Vazyme, Nanjing, China) on a Step One Plus instrument (ABI). Briefly, the procedure involved an initial reverse transcription at 55 °C for 15 min, followed by pre-denaturation at 95 °C for 30 s. The qPCR reactions were run for 40 cycles with denaturation at 94 °C for 10 s, annealing, and extension at 60 °C for 30 s.

### 2.2. Phylogenetic Analysis and Recombination Analysis

The reverse transcription and amplification of the RNAs in positive samples were performed by using the HiScript II One-Step RT-PCRKit (DyePlus) following the manufacturer’s instructions (Vazyme, Nanjing, China). Briefly, the procedure involved an initial reverse transcription at 45 °C for 25 min, followed by predenaturation at 94 °C for 5 min. The PCR reactions were run for 30 cycles with denaturation at 94 °C for 30 s, annealing at 55 °C for 30 s, extension at 72 C for 1, 2 min (for VP7 and VP4 genes), and final extension at 72 C for 5 min. The genome of RVA was amplified using universal primers (Table S1) designed by our laboratory. The amplified RVA gene was subsequently purified and Sanger sequenced (Huada Gene, Shenzhen, China). The obtained RVA sequences were compared using DNAStar 6.0 (DNASTAR, Madison, WI, USA). These rotavirus sequences were then compared with sequences in the GenBank database. The genetic distance between the isolates and reference strains was determined by genetic evolution analysis of nucleotides. The phylogenetic tree was constructed by the neighbor-joining method with 1000 bootstrap replicates via MEGA 11.0 (Tamura K, Stecher G, and Kumar S 2021). Simultaneously, the recombination detection program RDP4.101 (Simmonds and Welch 2006) was utilized to identify recombination events in the whole genome of the rotavirus; the characterization of recombination depends on the identification of recombination events through the detection of changes in phylogenetic tree topology.

### 2.3. Virus Isolation and Identification

The supernatants from the positive clinical samples were filtered and then treated with 30 µg/mL trypsin for 1 h at 37 °C. The activated supernatant was subsequently added to MA104 cells (obtained from ATCC), which formed a dense monolayer. After 1 h, the viral mixture was aspirated and discarded. Next, DMEM containing a final concentration of 4 μg/mL trypsin was added. The cytopathic effect (CPE) was observed 24 h post-infection.

After approximately 20 h of RVA infection, the MA104 cells exhibit 60–70% CPE. An indirect immunofluorescence assay was conducted on the cells that exhibited lesions. For fixation, the supernatant was discarded, the wells were washed and 100% methanol (Biosharp, Hefei, China) precooled at −20 °C was added for fixation at the same temperature for 15 min. Following fixation, the blocking solution was incubated for 1 h. In the antibody incubation step, a diluted RVA VP7 (G9) protein monoclonal antibody (Biosharp, Hefei, China) was added. The cells were washed and then reacted with the FITC-labeled goat anti-mouse antibody (Biosharp, Hefei, China) for 1 h. The cells were washed and incubated with DAPI at 37 °C for 15 min. The monolayer of cultured cells was washed three times with PBST.

### 2.4. Western Blotting

Rotavirus-infected MA104 cells were lysed for 30 min with lysis solution (Biosharp, Hefei, China). Separation was achieved through electrophoresis, immediately followed by transfer onto a polyvinylidene difluoride membrane (Biosharp, Hefei, China). Following membrane transfer, the membranes were blocked with 5% BSA for 2 h. The RVA VP7 (G9) protein monoclonal antibody (Biosharp, Hefei, China), which was diluted in 5% BSA prepared in PBST, was incubated at 4 °C overnight. The membrane incubated with an HRP-labeled sheep anti-rabbit antibody (Biosharp, Hefei, China) for 2–3 h. After cleaning, the color developing solution was subsequently added to the dish, and the membrane was observed for well-exposed images, which were subsequently analyzed.

### 2.5. Electron Microscopy

The cells were inoculated with a virus solution until lesions were produced and then washed twice with PBS. Next, 2.5% glutaraldehyde at room temperature was added for fixation. The fixed samples were subsequently embedded and then processed for sectioning. Following sectioning, haematoxylin and eosin (HE) staining analysis was performed to observe the pathological changes in big mouse tissues. The sections were subjected to staining and observed under a transmission electron microscope (Servicebio, Wuhan, China).

### 2.6. Plaque Assay

Once the MA104 cells formed a monolayer, the culture medium was aspirated. A virus solution was prepared and subjected to tenfold gradient dilution to obtain 10 concentrations. An appropriate volume of virus solution was allowed to adsorb for 1 h at 37 °C. Next, a 2% low melting point agarose solution was added. After cooling, the agarose solidified into a covering layer, and the plates were placed in a carbon dioxide incubator at 37 °C for cultivation. The cell lesions were observed daily, and a second covering was applied when obvious lesions appeared. The mixture prepared using the above method was supplemented with neutral red to achieve a concentration of 0.002%. The plaque size was determined by measuring the average diameter of the plaques.

### 2.7. Pathogenicity Studies in Rats

Ten-day-old Sprague–Dawley (SD) rats were chosen for the RVA infection test to investigate virus pathogenicity. The rats were procured from the Laboratory Animal Center of Huazhong Agricultural University (Wuhan, China), and all experimental procedures received approval from the Ethics and Welfare Committee (HZAURA-2024-0023). Several strains of RVA were isolated from samples and cultured in cells. RVA strains exhibiting high viral copy numbers were chosen for high-dose gavage (7 × 10^8^ PFU) in rats, with 5 rats assigned per strain. The control rats received DMEM via oral gavage, and the rats were all monitored daily for any clinical signs. The intestines were harvested from each rat group at 4 days post infection (dpi) to assess the viral load. The pathogenicity of RVA was further investigated by selecting strains that demonstrated significant effects on viral colonization on the basis of the small intestinal viral load. Each strain was divided into three groups: a high-dose group infected with RVA at 7 × 10^8^ PFU, a medium-dose group infected with RVA at 7 × 10^7.5^ PFU, and a low-dose group infected with RVA at 7 × 10^7^ PFU. The control rats received DMEM via oral gavage, while the rats were monitored daily for any clinical signs. Hearts, livers, spleens, lungs, kidneys, and intestines were harvested from each rat group at 4 days post infection (dpi) to assess the viral load in each organ. Immediate treatment is necessary for rats that succumb to RVA. The tissues were subsequently fixed in a 4% paraformaldehyde solution for pathological examination.

### 2.8. Immunohistochemistry

The tissue sections were fixed in anhydrous ethanol at 4 °C overnight, rinsed with running water for 1 min and washed with a phosphate buffer solution (pH 7.3) for 5 min. The RVA VP7 (G9) protein monoclonal antibody (Biosharp, Hefei, China) was diluted with Antibody Diluent at a dilution ratio of 1:1000 and then added dropwise to the sample, followed by overnight incubation at 4 °C and washing with phosphate buffer solution three times for 5 min each. The samples were incubated with the HRP-labeled goat anti-mouse antibody (Biosharp, Hefei, China), added dropwise to the samples at a 1:1000 dilution. DAB was used for color development for 30 s. Hematoxylin treatment was performed for 2 min, followed by washing the plates with running water for 10 min, dehydration with 75%, 85%, 95%, and 100% ethanol for 5 min each, treatment with xylene for 5 min, and sealing with neutral gum. The samples were stained with a dark brown color to indicate antibody-positive expression, whereas no specific coloration was observed for antibody-negative expression.

## 3. Results

### 3.1. Positive Rate of Swine Enteric Viruses

Enteral samples collected from December 2021 to February 2024 were subjected to a quantitative real-time RT-PCR assay to detect porcine diarrhea viruses. Among the 2242 diarrhea samples, 943 tested positive for RVA, resulting in a positive rate of 42.0%. Among the RVA-positive samples, approximately 11.5% (221/2242) were coinfected with PEDV, followed by 2.94% (66/2242) with PDCoV and 1.29% (41/2242) with TGEV. Coinfections with PEDV, RVA, TGEV, and PDCoV were extremely rare [20].

**Figure 1:**
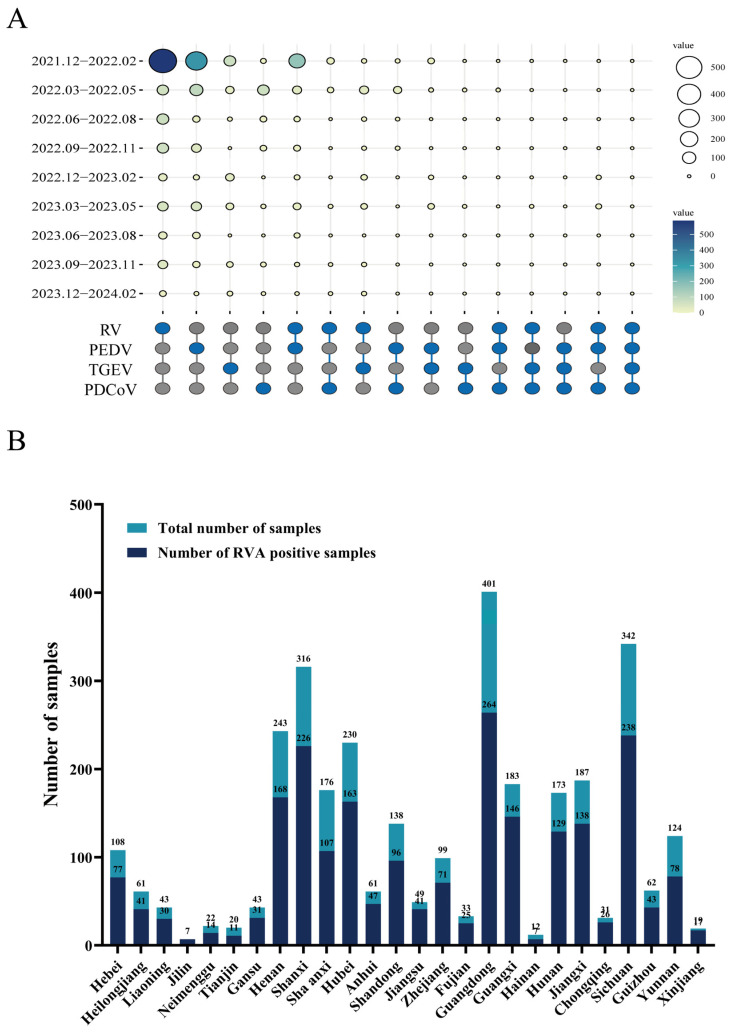
Histogram of porcine rotavirus A infection in China. (**A**) The coinfection rate of swine enteric viruses was analyzed in intestinal tissue samples collected from 2022 to 2024. A diagram was created for 2242 samples, depicting the frequency of four prevalent swine diarrhea viruses: PEDV, TGEV, RVA, and PDCoV. The color shades and size of circles in the upset plot indicate the sample size per season. (**B**) The positive rate of RVA in samples collected in different provinces from 2021 to 2024. The *X*-axis indicates the province, and the *Y*-axis indicates the RVA positive number.

Additionally, a high rate of RVA positivity was maintained in each season (Figure 1A). This study included data from 26 provinces, primarily in the eastern, southern, and southwestern regions. The detection results revealed that the prevalence of RVA infection in central China (Gansu, Henan, Hubei, and Shanxi) was 45.03%. The rates of RVA-positive cases in southern (Guangdong, Guangxi, Hainan, Hunan, Jiangxi) and southwestern (Sichuan, Guizhou, Yunnan, Chongqing) China were approximately 39.8% and 44.9%, respectively (Figure 1B and Figure S1).

### 3.2. Isolation and Identification of RVA

**Figure 2:**
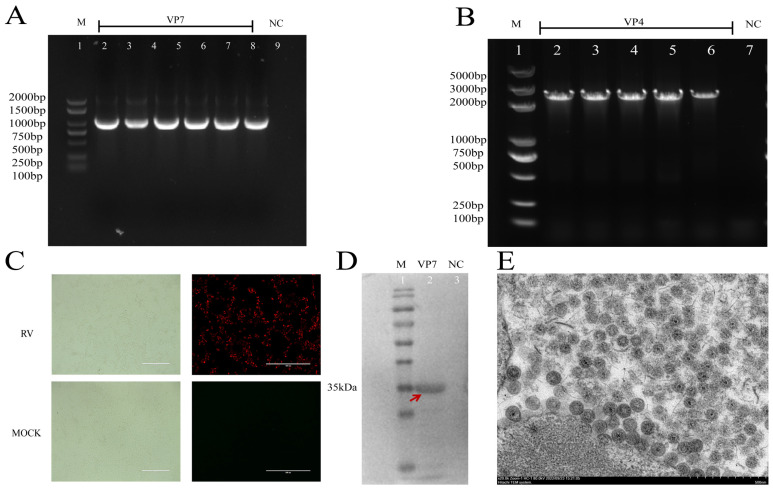
Isolation and identification of Porcine Rotavirus A (RVA). (**A**,**B**) PCR detection of RVA in swine enteric samples. ddH2O was used as a negative control (Lane 9). (**C**) Cytopathic effects (CPEs) of MA104 cells infected with RVA isolates observed under a microscope (200×); scale bar equals 400 μm. Indirect immunofluorescence analysis (IFA) specific to RVA isolates showing red fluorescence in MA104 cells. The scale bar represents 400 µm. (**D**) Western blotting was used to detect MA104 cells infected with an RVA strain, and an RVA VP7 monoclonal antibody was used for identification. (**E**) Transmission electron microscopy image of an RVA virion. The scale bar represents 500 nm.

RVA was detected as a single positive by RT-PCR, as shown in Figure 2A,B. RVA isolates were identified on the basis of cytopathic effects (CPEs), such as distinct shrunken, seine, and syncytium effects, and immunofluorescence (IFA) was performed using a monoclonal antibody targeting the RVA VP7 (G9) protein (Figure 2C) and purification via plaque. The virus was identified through Western blotting, which revealed bands at 37.2 kDa, with an RVA VP7 monoclonal antibody employed for detection. (Figure 2D). A few round wheel-like particles were observed in the endoplasmic reticulum (ER) of the MA104 cells, and the virion was spherical with a diameter of 100 nm, which is consistent with the morphology of an RVA virion (Figure 2E).

### 3.3. Phylogenetic Analysis of the VP4 and VP7 Genes

**Figure 3:**
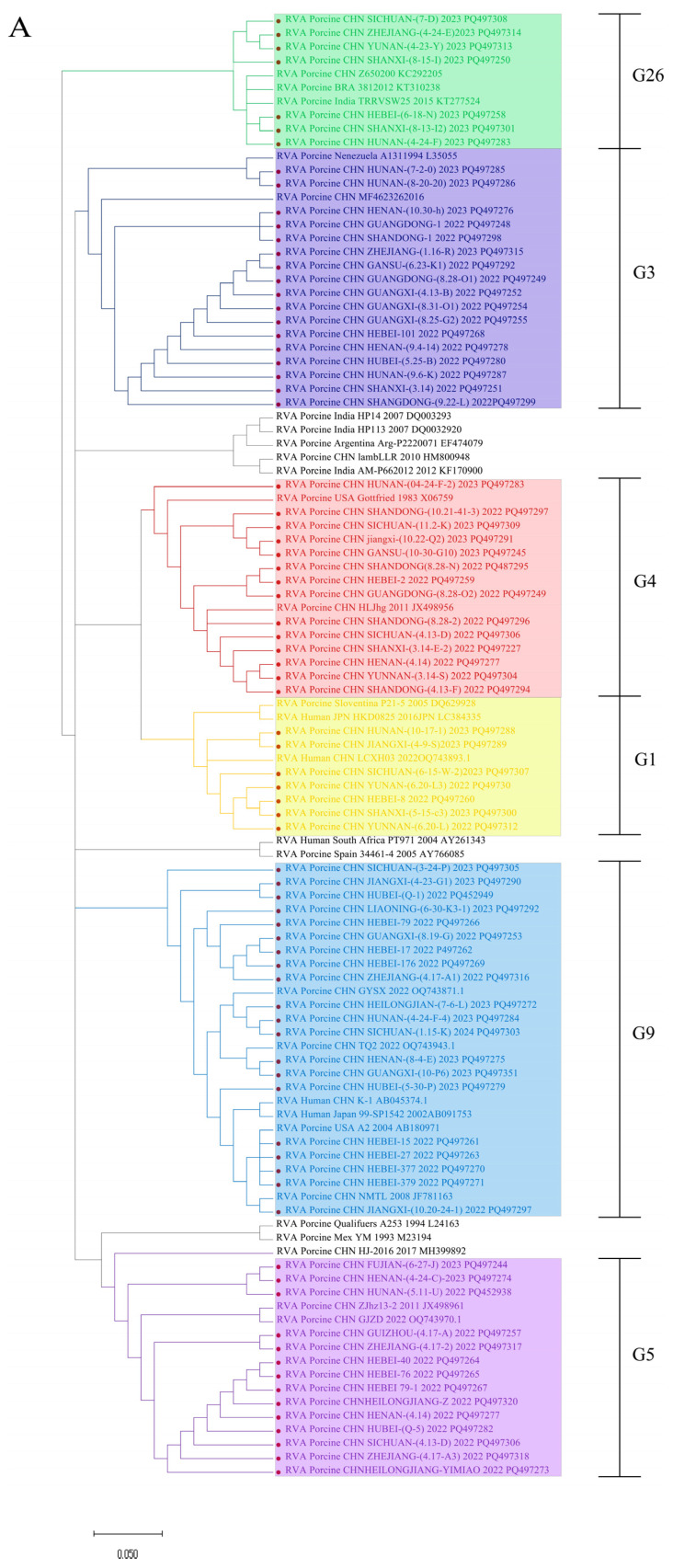

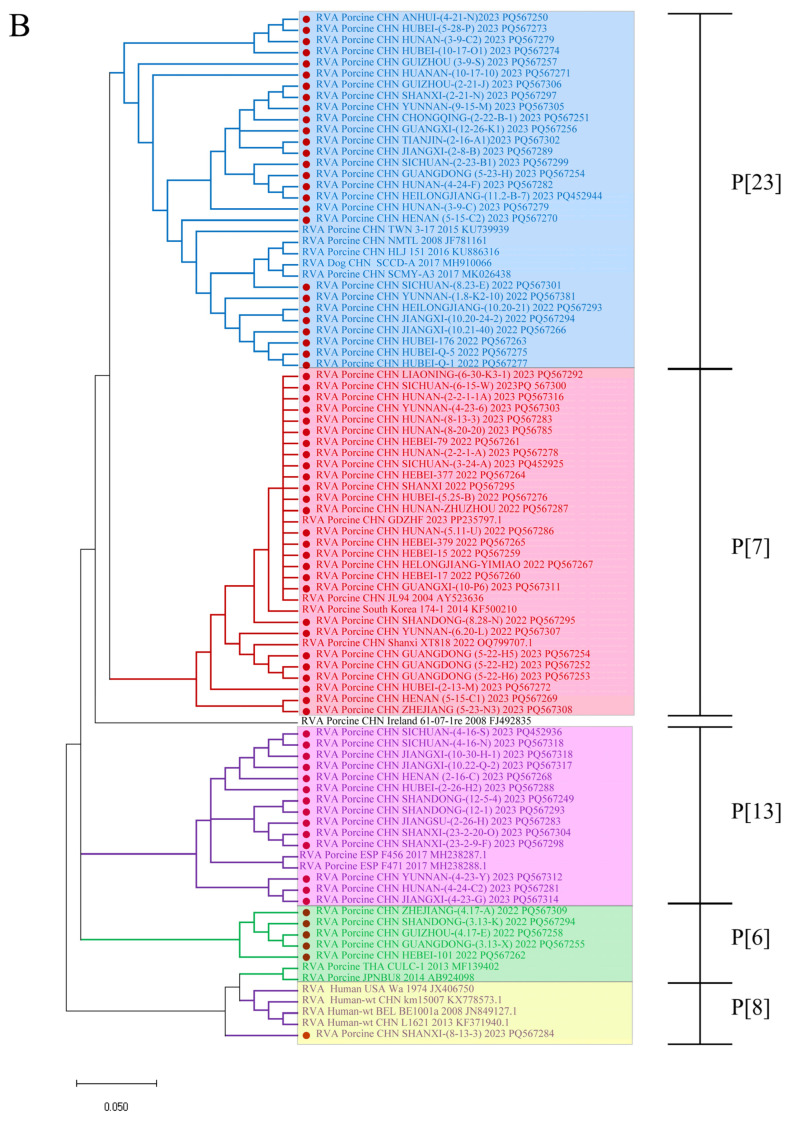

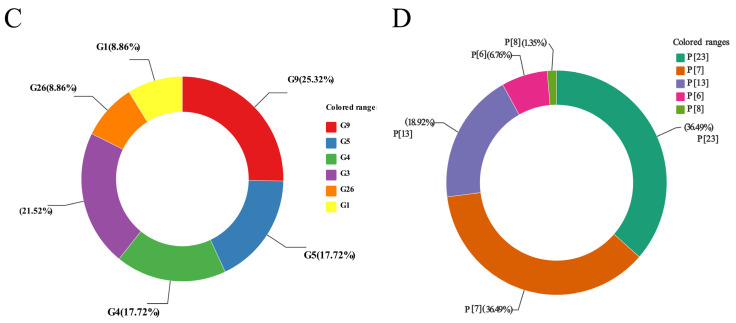
Phylogenetic analysis of the VP4 and VP7 genes of the RVA strains collected in this study. (**A**) Phylogenetic analysis of VP7 proteins from 79 RVA strains. (**B**) Phylogenetic analysis of VP4 proteins from 74 RVA strains. (**C**) Proportion of different G genotypes of rotavirus. (**D**) Proportion of different P genotypes of rotavirus. In the trees, the position of the isolated RVA strain is shown by a red circle.

The phylogenetic relationships of the RVA strain isolates in this study were analyzed by aligning their VP4 and VP7 nucleotide sequences with reference sequences from GenBank to construct a phylogenetic tree. The results are shown in Figure 3. Six genotypes of 79 VP7 were identified for RVAs, with G9 (25.32%, 20/79), G5 (17.72%, 14/79), G4 (17.72%,14/79), and G3 (21.52%, 14/79) being the predominant types. Additionally, G26 (8.86%, 7/79), G1 (8.86%, 7/79), and other types were detected (Figure 3A,C). The predominant genotypes of 74 VP4 were P23 and P7 (36.49%, 27/74; 36.49%, 27/74), followed by P13 (18.92%, 14/74), P6 (6.76%, 5/74), and P8 (1.35%, 1/79) (Figure 3B,D). This finding suggests great diversity and a complex background of RVA epidemiology.

### 3.4. Isolation, Sequencing, Homology, and Recombination Analysis of the Predominant Strain

**Figure 4:**
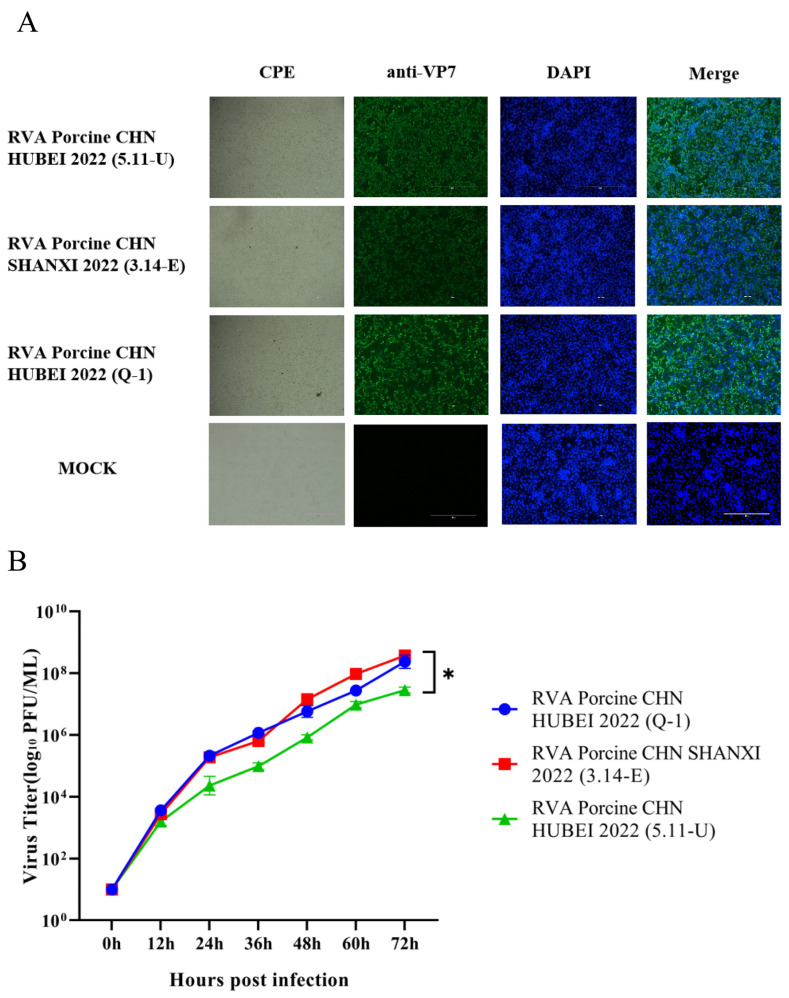
Characteristics of epidemics of candidate RVAs. (**A**) Indirect immunofluorescence staining was performed to detect the RVA VP7 protein in infected MA104 cells. The VP7 protein is stained green, while DAPI stains the nuclei blue. Fluorescence microscope images were taken, with a scale bar representing 400 µm. (**B**) Viral titers in the supernatants from various infection times were assessed using the PFU method and are reported as the means ± SEMs. *, *p* < 0.05.

During the virus isolation and culture process, we isolated three highly virulent RVA strains: RVA Porcine CHN HUBEI 2022 (Q-1), RVA Porcine CHN SHANXI 2022 (3.14-E), and RVA Porcine CHN HUBEI 2022 (5.11-U). We demonstrated the colonization of RVA Porcine CHN HUBEI 2022 (Q-1), RVA Porcine CHN SHANXI 2022 (3.14-E), and RVA Porcine CHN HUBEI 2022 (5.11-U) in the cytoplasm of MA104 cells by IFA (Figure 4A). Among them, replication kinetics were observed in cultured MA104 cells at 12, 24, 35, 48, 60, and 72 h post infection (Figure 4B). The proliferative capacity of RVA Porcine CHN HUBEI 2022 (5.11-U) was lower than that of RVA Porcine CHN HUBEI 2022 (Q-1) and RVA Porcine CHN SHANXI 2022 (3.14-E) (Figure 4B).

**Figure 5:**
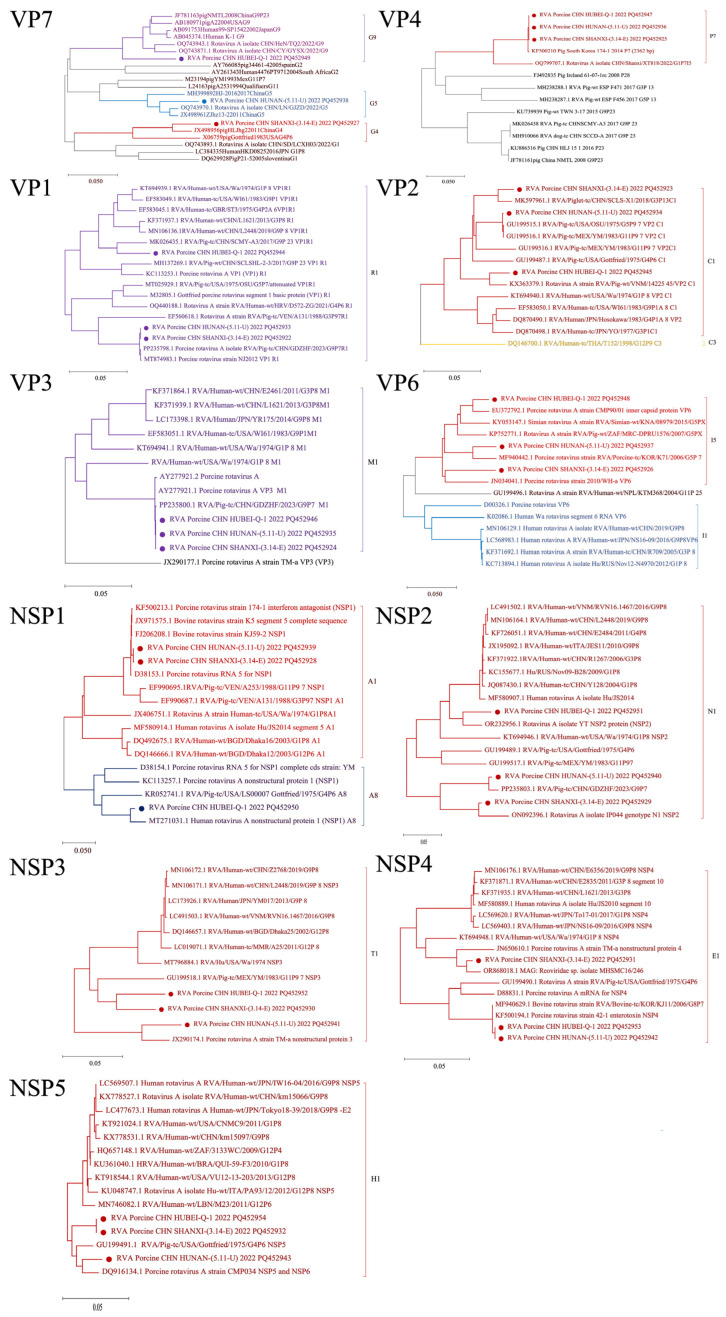
The complete nucleotide sequence of the RVA gene was utilized to construct a phylogenetic tree. In the trees, the positions of RVA Porcine CHN HUBEI 2022 (Q-1), RVA Porcine CHN SHANXI 2022 (3.14-E), and RVA Porcine CHN HUBEI 2022 (5.11-U) are shown by circles. Scale bar, 0.05 substitutions per nucleotide.

The VP7-VP4-VP6-VP1-VP2-VP3-NSP1-NSP2-NSP3-NSP4-NSP5 genotypes of RVA Porcine CHN HUBEI 2022 (Q-1), RVA Porcine CHN SHANXI 2022 (3.14-E), and RVA Porcine CHN HUBEI 2022 (5.11-U) strains were assigned as the G9-P[7]-I5-R1-C1-M1-A8-N1-T1-E1-H1, G4-P[7]-I5-R1-C1-M1-A1-N1-T1-E1-H1, and G5-P[7]-I5-R1-C1-M1-A1-N1-T1-E1-H1 genotypes by nucleotide sequencing and phylogenetic analysis. The RVA Porcine CHN HUBEI 2022 (Q-1) strain differed from the RVA Porcine CHN SHANXI 2022 (3.14-E) and RVA Porcine CHN HUBEI 2022 (5.11-U) strains in only the VP7 and NSP1 genes, as it was identified as the G9 and A8 genotype. Conversely, the VP7 genes of the Porcine CHN SHANXI 2022 (3.14-E)/HUBEI 2022 (5.11-U) strains were assigned as the G4 and G5 genotypes. Additionally, the NSP1 genes of the Porcine CHN SHANXI 2022 (3.14-E)/HUBEI 2022 (5.11-U) strains were identified as the A1 genotype, whereas the other nine genes were similar to those of Porcine CHN HUBEI 2022 (Q-1) (Figure 5).

**Figure 6:**
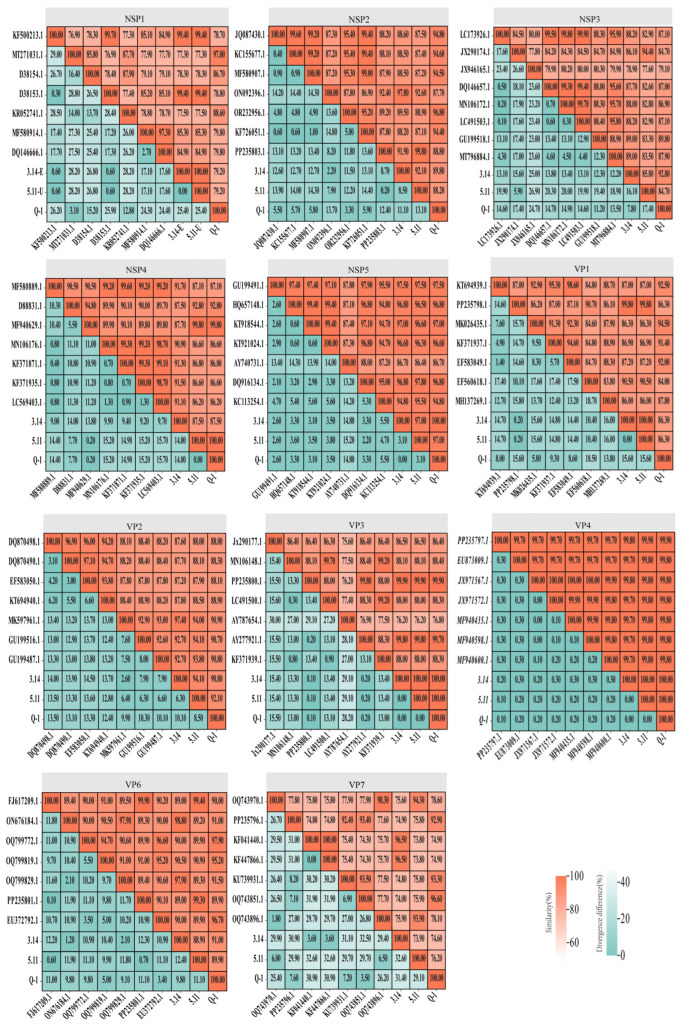
Percent sequence identity of the whole genes of the RVAs strains.

The results are shown in Figure 6. The sequences of VP1, VP2, VP3, VP4, VP6, VP7, and NSP1-NSP5 were analyzed for RVA Porcine CHN HUBEI 2022 (Q-1), RVA Porcine CHN SHANXI 2022 (3.14-E), and RVA Porcine CHN HUBEI 2022 (5.11-U) obtained in this study, along with other RVA strains from GenBank. The results in Figure 6 demonstrate that the VP7 genes of RVA Porcine CHN HUBEI 2022 (Q-1), RVA Porcine CHN SHANXI 2022 (3.14-E), and RVA Porcine CHN HUBEI 2022 (5.11-U) presented the highest similarity to OQ743851.1 at 96.6%, KF447866.1 at 96.5%, and OQ743970.1 at 94.3%, respectively, and were classified as belonging to the G9, G4, and G5genotypes, as shown in Figure 5. The VP4 gene consists of 2331 nucleotides, encoding 777 amino acids, and exhibited the highest similarity to PP235797.1 at 99.9%, 99.8%, and 99.9% for RVA Porcine CHN HUBEI 2022 (Q-1), RVA Porcine CHN SHANXI 2022 (3.14-E), and RVA Porcine CHN HUBEI 2022 (5.11-U), respectively. Similarly, the VP6 gene comprises 1194 nucleotides, encoding 398 amino acids, and showed the highest similarity to OQ799772.1 at 97.9%, ON676184.1 at 98.8%, and FJ617209.1 at 99.4% for the same strains. Additionally, the VP1 gene consists of 3267 nucleotides, encoding 1089 amino acids, and it exhibited the highest similarity to MK026435.1 at 94.5%, PP235798.1 at 99.8%, and PP235798.1 at 99.8% for RVA Porcine CHN HUBEI 2022 (Q-1), RVA Porcine CHN SHANXI 2022 (3.14-E), and RVA Porcine CHN HUBEI 2022 (5.11-U), respectively. The VP2 gene comprises 2673 nucleotides encoding 891 amino acids. The VP2 genes of RVA Porcine CHN HUBEI 2022 (Q-1), RVA Porcine CHN SHANXI 2022 (3.14-E), and RVA Porcine CHN HUBEI 2022 (5.11-U) exhibited the highest similarity to MK597961.1 at 90.9%, MK597961.1 at 97.4%, and GU199516.1 at 94.1%, respectively. Similarly, the VP3 gene comprises 2508 nucleotides, encoding 836 amino acids, and showed the highest similarity to PP235800.1 at 99.9%, 99.9%, and 99.9% for the same strains. The NSP1 gene comprises 1461 nucleotides, encoding 487 amino acids, and exhibited the highest similarity to MT271031.7 at 97.0%, D38153.1 at 99.4%, and KF500213.1 at 99.4%, respectively, and belonged to the A8, A1, and A1 serotypes, as depicted in Figure 5. Similarly, the NSP2 gene comprises 954 nucleotides, encoding 318 amino acids, and showed the highest similarity to OR232956.1 at 96.8%, ON092396.1 at 97.8%, and PP235803.1 at 99.8% for the same strains. The NSP3 gene comprises 942 nucleotides, encoding 314 amino acids, and exhibited the highest similarity to GU199518.1 at 89.8%, MT796884.1 at 89.0%, and JX290174.1 at 94.4% for the same strains. Similarly, the NSP4 gene comprises 528 nucleotides, encoding 176 amino acids, and it showed the highest similarity to MF940629.1 at 99.8%, MF580889.1 at 91.7%, and MF940629.1 at 99.8% for RVA Porcine CHN HUBEI 2022 (Q-1), RVA Porcine CHN SHANXI 2022 (3.14-E), and RVA Porcine CHN HUBEI 2022 (5.11-U). The NSP5 gene contains 594 nucleotides encoding 198 amino acids. The NSP5 genes of RVA Porcine CHN HUBEI 2022 (Q-1), RVA Porcine CHN SHANXI 2022 (3.14-E), and RVA Porcine CHN HUBEI 2022 (5.11-U) were most closely related to GU199491.1 at 97.5%.

**Figure 7:**
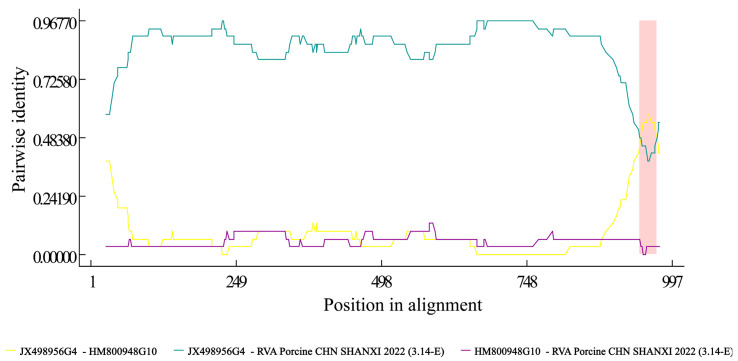
Recombination analysis of RVA Porcine CHN SHANXI 2022 (3.14-E) VP7 gene with other RVA strains. The comparison of the recombinant major parent and the recombinant minor parent is indicated by green and yellow lines, respectively, and the red areas indicate the presence of reorganization.

Recombination analysis of the VP7 gene was performed using RDP4.101 software, which utilized both the RVA strains obtained in this study and 45 reference strains. The analysis revealed potential recombination breakpoints in the RVA Porcine CHN SHANXI 2022 (3.14-E) strain at nucleotide positions 940 to 978, as determined by alignment with the VP7 gene, in comparison to two parental RVA strains (JX498956 and HM800948.1). More specifically, nucleotide positions 940 to 978 of the VP7 gene in the RVA Porcine CHN SHANXI 2022 (3.14-E) strain were predicted to constitute a recombinant fragment arising from recombination events involving JX498956 (major parent, G4) and HM800948.1 (minor parent, G10), suggesting the occurrence of genetic recombination in this region. However, no recombination was detected in any other viral genes, as illustrated in Figure 7.

### 3.5. Pathogenicity in Rats

**Figure 8:**
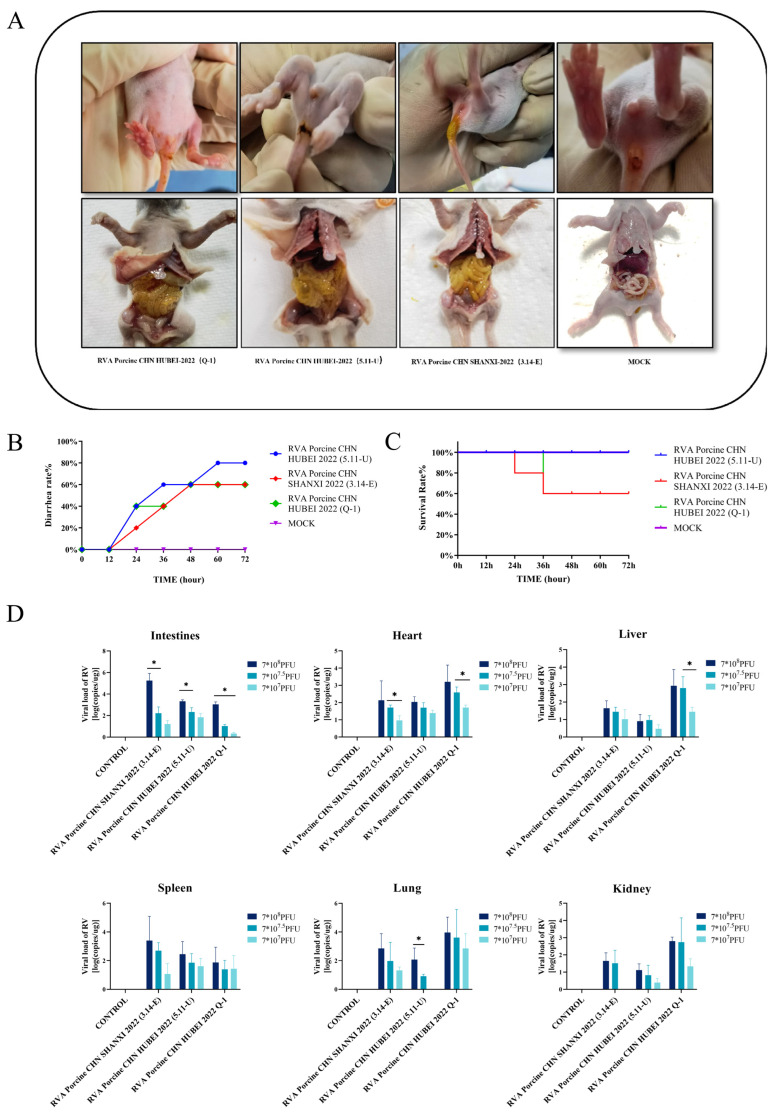
Evaluation of the pathogenicity of RVAs RVA Porcine CHN HUBEI 2022 (Q-1), RVA Porcine CHN SHANXI 2022 (3.14-E), and RVA Porcine CHN HUBEI 2022 (5.11-U). (**A**) Representative clinical signs and gross examination of challenged rats. (**B**) Diarrhea rate in the rats. (**C**) Survival rate of the rats. (**D**) Each tissue from infected rats—intestine, heart, liver, spleen, lung and kidney—was tested using quantitative real-time RT-PCR. *, *p* < 0.05.

Ten-day-old rats were selected to evaluate the pathogenicity of the RVA strains RVA Porcine CHN HUBEI 2022 (Q-1), RVA Porcine CHN SHANXI 2022 (3.14-E), and RVA Porcine CHN HUBEI 2022 (5.11-U) by oral inoculation with the respective strains or DMEM. The rats in the experimental group presented severe diarrhea symptoms at 1 dpc, whereas those in the control group remained normal (Figure 8A and Figure S2). The rates of diarrhea at 72 h for RVA strains RVA Porcine CHN HUBEI 2022 (Q-1), RVA Porcine CHN SHANXI 2022 (3.14-E), and RVA Porcine CHN HUBEI 2022 (5.11-U) were 60%, 60%, and 80%, respectively (Figure 8B). Among them, the RVA strains RVA Porcine CHN HUBEI 2022 (Q-1) and RVA Porcine CHN SHANXI 2022 (3.14-E) exhibited mortality in rats at 24 h and 36 h after challenge, with a potential mortality rate of up to 40% at 72 h (Figure 8C). At necropsy, transparent, thin-walled, gas-distended intestines filled with yellow contents were observed in the experimental group of challenged rats (Figure 8A). Tissues, including intestines, hearts, livers, spleens, lungs, kidneys, and brains, were collected from rats infected with the RVA Porcine CHN HUBEI 2022 (Q-1), RVA Porcine CHN SHANXI 2022 (3.14-E), and RVA Porcine CHN HUBEI 2022 (5.11-U) strains for virus copy number determination upon the onset of significant clinical symptoms. RVA presented the highest viral copy number in the intestine, with proliferation also observed in the spleen and lungs. RVA was not detected in the control rat tissues (Figure 8D). With increasing doses, the copy number of virions in rat tissue increased, and there was a dose-dependent trend (Figure 8D). These data suggest that the RVA Porcine CHN HUBEI 2022 (Q-1), RVA Porcine CHN SHANXI 2022 (3.14-E), and RVA Porcine CHN HUBEI 2022 (5.11-U) strains are significantly pathogenic in rats.

### 3.6. Histopathology of the Intestines

**Figure 9:**
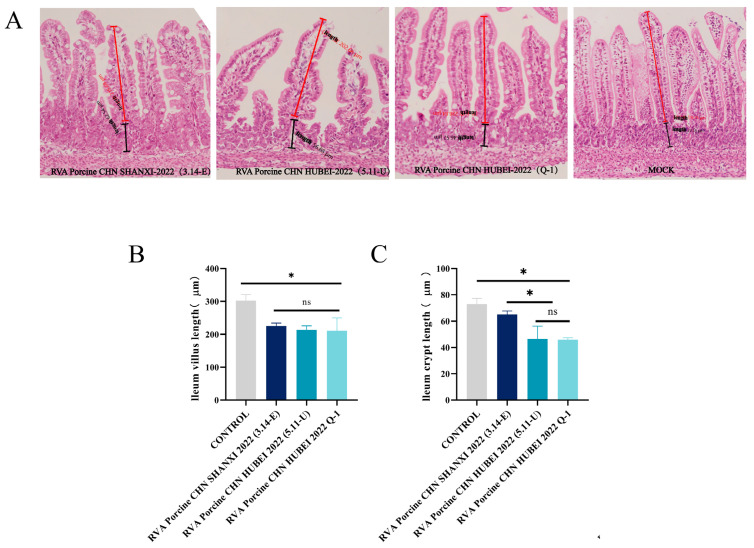
Pathological study of the intestinal tract in rats with RVA. (**A**) Histopathological examination of the small intestine. (**B**,**C**) Quantification of small intestinal villus length and intestinal crypt length. The data are presented as the means ± SDs. *, *p* < 0.05; ns, *p* > 0.05 (ANOVA).

Histopathological observation allowed for a more accurate determination of the pathogenicity of RVA Porcine CHN HUBEI 2022 (Q-1), RVA Porcine CHN SHANXI 2022 (3.14-E), and RVA Porcine CHN HUBEI 2022 (5.11-U) in rats. Microscopic examination revealed evident histologic lesions characterized by severe and extensive scattered, disrupted, and fused villi throughout the small intestine in the groups challenged with RVA Porcine CHN HUBEI 2022 (Q-1), RVA Porcine CHN SHANXI 2022 (3.14-E), and RVA Porcine CHN HUBEI 2022 (5.11-U) (Figure 9A). Both the length of the small intestinal villi and the depth of the crypts were significantly lower in the challenged group than in the control group (Figure 9B,C).

## 4. Discussion

As leading causes of swine diarrhea, PEDV, RVA, PDCoV, and TGEV are frequently encountered on pig farms worldwide [22,23]. Among these pathogens, PEDV has the most significant effect on piglet diarrhea. Recent studies have shown a decreasing trend in the positive detection rate of PEDV since 2018. This decline can be attributed partly to the protective effect of the GII-b strain vaccine currently available on the market. In contrast, the prevalence of RVA has shown the opposite trend, with its positive detection rate increasing from 4% to 10.45% between 2018 and 2021 [2,23,24]. It has been reported that the RVA positivity rate in coastal provinces such as Zhejiang, Shandong, and Jiangsu was only 7% from 2013 to 2019 [25]. This study investigated the infection rate of RVA through a comprehensive analysis of 2242 diarrhea-related samples collected from Chinese pig farms in 2021 and 2024. The data revealed an RVA-positive rate of almost 42% in the pig farms where samples were analyzed. Further analysis revealed that the PEDV and RVA coinfection rates were the highest, at 11.5%, in this study. The positive rate of RVA and the positive rate of RVA in each region increased significantly compared with those before 2020 [26]. The commercialized RVA vaccine in China only targets the G5 strain, while the results of this study revealed that the dominant RVA genotype in some areas of China is the G9 strain. It is not sufficiently protective for swine because of poor cross-protection between different genotypes [27]. In addition, RVA often presents as a latent infection in adult pigs, which can become symptomatic when external factors change or when immunity decreases.

To distinguish RVA genotypes effectively, a two-class nomenclature is commonly employed to classify the G and P genotypes of strains via the VP4 and VP7 genes. Owing to the high genetic diversity of these genome segments, at least 27 rotavirus G genotypes and 37 P genotypes have been identified [9]. Commonly occurring G genotypes include G1–G6, G9, and G11, whereas prevalent P genotypes include P[6]–P[8], P[11], P[13], P[23], and P[32]. The predominant G genotypes reported to confirm transmission in domestic pigs are G9, G5, and G3, accounting for 68.7% of cases. The predominant P genotypes are P[13] and P[23], accounting for 82.1% of cases, with the combination of G5P[7] genotypes having a significant predominance in domestic pigs worldwide [28].

In this study, 79 RVA strains from pig farms in certain regions of China were identified by G genotype assignment, and 74 RVA strains were identified by P serotype assignment. The G9 genotype was the dominant G genotype, whereas the P[7] and P[23] genotypes were the dominant P genotypes. Among them, the dominant genotypes of porcine rotavirus G genotypes underwent changes, with a decrease in the proportion of G5 genotypes and an increase in the proportion of G9 genotypes. The VP7 gene serves as the primary target of neutralizing antibodies, and a change in its genotype results in a reduced cross-protection rate between strains. This outcome was predictable because of increased animal movements among large-scale pig farms, leading to the high genetic diversity of RVA strains. The limited number of samples in this study cannot accurately reflect the incidence of RVA in some areas of China, but it still demonstrates the presence of RVA with multiple genotype combinations. As the prevalence of RVA increases, its genetic diversity also increases, leading to more diverse combinations of G and P serotypes. Therefore, continuous monitoring of RVA genetic variation can inform vaccine development and disease prevention and control strategies.

In our study, the PCR, IFA, WB, and TEM results confirmed the successful isolation of RVA. Eleven high-virulence RVA strains of different serotypes were orally administered to 10-day-old rats at high doses. After the experiment, the viral load in the small intestines of the rats was assessed to identify the predominant strains. Eleven RVA strains exhibited varying degrees of colonization in the rat intestines, with three strains having high copy numbers ranging from 1 × 10^2.16^–1 × 10^5.08^. Previous reports indicate that cross-protection between different genotypes of RVA strains is limited [29,30]. RVA Porcine CHN HUBEI 2022 (Q-1), RVA Porcine CHN SHANXI 2022 (3.14-E), and RVA Porcine CHN HUBEI 2022 (5.11-U) were identified as predominant strains on the basis of viral copy values and the prevailing genotypes of RVA. Homology and phylogenetic analyses revealed that the isolates belonged to the G9P7, G4P7, and G5P7 genotypes. Whole-genome sequence analysis of RVA strains is crucial for comprehending strain diversity [31]. Therefore, whole-genome sequencing contributes to understanding RVA prevalence in Chinese pigs and developing vaccines targeting dominant genotypes. In this study, whole-genome sequence analysis was conducted on the porcine RVA strains, RVA Porcine CHN HUBEI 2022 (Q-1), RVA Porcine CHN SHANXI 2022 (3.14-E), and RVA Porcine CHN HUBEI 2022 (5.11-U). We found that these RVA strains exhibit the genotypes G9-P[7]-I5-R1-C1-M1-A8-N1-T1-E1-H1, G4-P[7]-I5-R1-C1-M1-A1-N1-T1-E1-H1, and G5-P[7]-I5-R1-C1-M1-A1-N1-T1-E1-H1. In this study, a similar genomic backbone was detected in three different serotypes of RVAs: I5-R1-C1-M1-A8/A1-N1-T1-E1-H1, which is similar to the genetic sequences known to be reported in porcine RVAs from other regions [22]. Homology analysis revealed that some strains presented greater similarity to human RVA strains than to porcine RVA strains. This result confirms the interspecies mobility of RVA strains between humans and animals and the transfer of genomic segments between species, emphasizing the dynamic interplay of RVA strains between humans and pigs. Studies have confirmed the prevalence of interspecies recombination and reassortment events between certain porcine-origin RVAs and human-origin RVAs [32]. In this study, a breakpoint in the VP7 gene of RVA was identified, suggesting that recombination events may contribute to the emergence of new viral genotypes. Recombination is recognized as a crucial mechanism in viral evolution and a potential driver of antigenic diversity [33,34].

In our study, porcine RVAs, including RVA Porcine CHN HUBEI 2022 (Q-1), RVA Porcine CHN SHANXI 2022 (3.14-E), and RVA Porcine CHN HUBEI 2022 (5.11-U), effectively replicated in the vital organs of rats, particularly the intestines, liver, spleen, lungs, and kidneys. These findings suggest that RVA may cause widespread systemic disease. Additionally, the detection of porcine rotaviruses in the lungs implies a potential mechanism of RVA transmission [35]. In the early stages of infection, diarrhea and death caused by dehydration were significant in rats infected with RVA Porcine CHN HUBEI 2022 (Q-1), RVA Porcine CHN SHANXI 2022 (3.14-E), and RVA Porcine CHN HUBEI 2022 (5.11-U). It has been demonstrated that day-old rats infected with rotavirus before 21 days of age exhibit varying degrees of diarrhea, the severity of which depends on the strain of virus used for inoculation [36]. Ciarlet M et al. reported that porcine rotavirus infects SD rats and induces significant pathological changes [37]. Histopathological examination revealed acute inflammation of the bile ducts due to rotavirus infection, along with inflammatory cell infiltration in the liver and lung parenchyma [38]. Rotavirus replication is also observed in macrophages within blood vessels and is accompanied by inflammatory cell accumulation [35]. Our histopathological examinations revealed that RVA caused significant damage to the alimentary tract and effectively proliferated in the intestines. The extent of the lesions also increased with increasing inoculum dose. These results indicate that RVA is highly pathogenic to rats. Currently, RVA is prevalent on pig farms, which makes it challenging to find antigen- and antibody-negative pigs for a model. Establishing a link between the porcine rotavirus rat model and a porcine rotavirus pig model could offer new insights and strategies for study.

The incidence of RVA has been increasing annually since 2015, and the prevalence of RVA has not been mitigated by widespread vaccination. This is due to substantial genetic variations among different genotypes of rotaviruses, leading to high susceptibility to cross-interchange. With an increasing number of reports about RVA, the virus has clearly become widespread on pig farms. Given the substantial genetic variation among different RVA serotypes, ensuring a match between the vaccine and circulating strains is crucial for effective prevention. Consequently, monitoring the virology of RVA and focusing on prevalent serotypes in China are essential, and the development of new vaccines containing multiple serotypes is crucial for preventing and controlling RVA.

## Data Availability

All data are available in this manuscript. Additional information can be obtained from the corresponding authors upon reasonable request. The nucleotide sequences were deposited in GenBank under the accession numbers PQ452944-PQ452954 (RVA Porcine CHN HUBEI 2022 (Q-1) VP1-NSP5), PQ452933-PQ452944 (RVA Porcine CHN HUBEI 2022 (5.11-U) VP1-NSP5), PQ452922-PQ452932 (RVA Porcine CHN SHANXI 2022 (3.14-E) VP1-NSP5), PQ452927 (VP7), PQ452938 (VP7), PQ497244-PQ457320 (VP7), PQ452925 (VP4), PQ452936 (VP4), PQ452947 (VP4), PQ567249-PQ567319 (VP4).

## Supplementary Materials

The following supporting information can be downloaded at: https://www.mdpi.com/article/10.3390/v16121842/s1, Table S1: Primes designed for PCR; Figure S1: Quantification of viral titers for RVA virus strains; Figure S2: Quantification of viral copy values of RVA in the rat intestine.
