# Supplementary material for: Molecular Characterization and Pathogenicity Analysis of Porcine Rotavirus A
Source: Viruses. 2024 Nov 27;16(12):1842. doi: 10.3390/v16121842 (PMC11680200; doi:10.3390/v16121842)
Supplement: Supplementary file 1 [file viruses-16-01842-s001.zip › viruses-3287153-supplementary.pdf]

Table S1 Primes and probes designed for real-time PT-QPCR and PCR

Virus	Prime/probe	Sequence (5’-3’)	Target gene	Size (bp)
RVA-VP1	Forward<br>Reverse	GGCTATTAAAGCTRTACAATGGGGAAG<br>GGTCACATCTAAGCGYTCTAAT	VP1	3303
RVA-VP2	Forward<br>Reverse	GGCTATTAAAGGYTCAATGGCGTAC<br>CATATCTCCACARTGGGGTTGG	VP2	2714
RVA-VP3	Forward<br>Reverse	GGCTWTTAAAGCARTATTAGTAGTG<br>GGTCACATCATGACTAGTGTG	VP3	2591
RVA-VP4	Forward<br>Reverse	GGCTATAAAATGGCTTCGC<br>GGTCACATCCTCTATAGAGCTCTC	VP4	2362
RVA-VP6	Forward<br>Reverse	GGCTTTWAAACGAAGTCTTC<br>GGTCACATCCTCTCACT	VP6	1356
RVA-VP7	Forward<br>Reverse	GGCTTTAAAAGAGAG<br>GGTCACATCATACAATTCT	VP7	1062
RVA-NSP1	Forward<br>Reverse	GGCTTTTTTTATGAAAAGTCTTGTG<br>GGTCACATTTTATGCTGCCTAG	NSP1	1566
RVA-NSP2	Forward<br>Reverse	GGCTTTTAAAGCGTCTCAGTC<br>GGTCACATAAGCGCTTTCTATTC	NSP2	1059
RVA-NSP3	Forward<br>Reverse	GGCTTTTAATGCTTTTCAGTGGTTG<br>GGTCACATAACGCCCCTATAG	NSP3	1074
RVA-NSP4	Forward<br>Reverse	GGCTTTTAAAAGTTCTGTTCCG<br>GGTCACATTAAGACCGTTCC	NSP4	750
RVA-NSP5	Forward<br>Reverse	GGCTTTTAAAGCGCTACAG<br>GGTCACAAAACGGGAGTG	NSP5	667

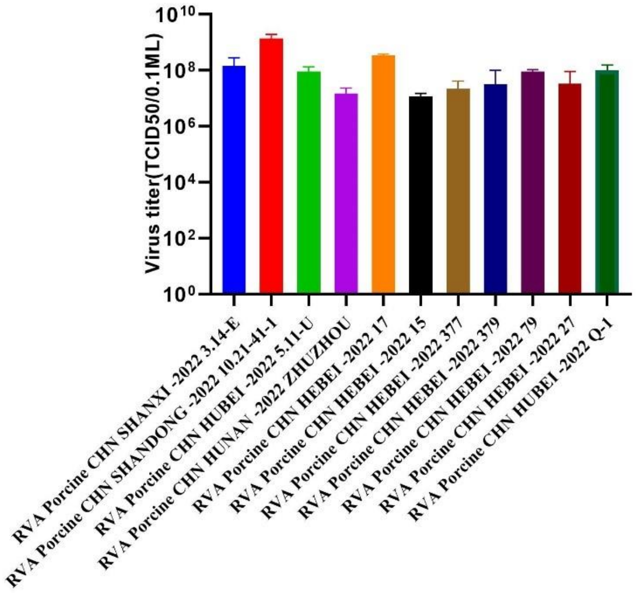

Figure S1. Quantification of viral titers for RVA virus strains.

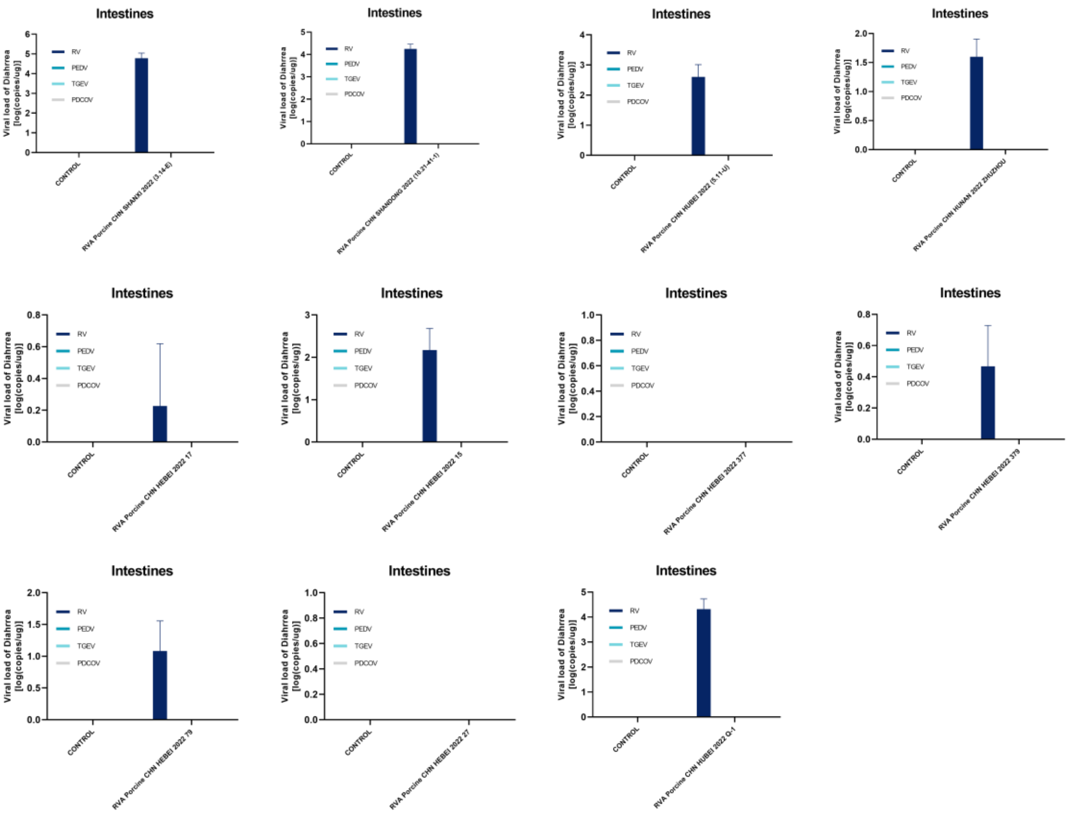

Figure S2. Quantification of viral copy values of RVA in the rat intestine
